# Fracture of the Acetabulum

**Published:** 1880-09

**Authors:** C. A. McBeth

**Affiliations:** Resident Physician Buffalo General Hospital


### Clinical Reports.

---

### FRACTURE OF THE ACETABULUM.—AUTOPSY.

REPORTED BY DR. C. A. McBETH, RESIDENT PHYSICIAN BUFFALO GENERAL HOSPITAL.

SERVICE OF DR. C. C. F. GAY.

Ella Smith entered the Hospital April 26, 1880. American. Single. Had been the mother of one or more children; contracted syphilis in September, 1879. Six hours previous to entering, fell from second-story window to pavement (13 feet), injuring her right hip. Dr. Frederick and myself gave her ether and made an examination, finding evertion of toe and crepitus There was no shortening, each limb measuring from anterior superior spinous process to internal malleolus 30½ inches. We did not consider a diagnosis of intra-capsular fracture positive, but as relief was afforded by extension we put on eight-pound pulleys and weight dressing.

April 27th Patient seen by Dr. Gay who made an examination, and carefully measured limbs, finding them same as night previous. She stated to-day that the evertion of toe was natural, having had it from childhood. Dressings by extension was therefore discontinued, thus allowing muscular action to shorten limb if there was a fracture.

May 5th. This is as early as it was deemed advisable to make further examination, on account of dysmenorrhœa from which she suffered. She was given ether, and it was found on measuring the limbs that the right was 31, and the left 30½ inches. A lengthening of half inch on the injured side. After examination Dr. Gay stated that the age of patient almost precluded possibility of intra-capsular fracture, and suggested the possibility of obturator-luxation. Consequently, in his opinion the difference of half inch was no evidence of fracture. Yet, as patient was relieved of pain by slight extension, he said we should again use the eight-pound weight.

For a time after this she was in a typhoid condition, and evidently suffering from septicæmia, having chills with subsequent fever, temperature reaching as high as 105° Fahrenheit, tenderness over caput coli and abdomen tympanitic. She sank gradually with very little pain till May 19th, 11.30 P. M., when she died. Rapid post-mortem changes.

Autopsy held 4.30 P. M., May 20th. Present—Drs. Gay, Diehl, Peterson and Frederick. In cutting down upon the hip found large quantity of pus evidently from the abdominal cavity as it could be forced through the opening in hip by pressure over abdomen. Vagina filled with pus. Found fracture of the os innominatum through the floor of the acetabulum as follows: one separating ilium from ischium and pubis, another separating ischium from pubis, both nearly in line of union, also fracture of ramus of pubis and a large triangular fragment from the upper border of the iliac surface of the acetabulum. The medulary substance of both femur and pelvic bones was somewhat broken down.

